# Three-Dimensional Multi-Task Deep Learning Model to Detect Glaucomatous Optic Neuropathy and Myopic Features From Optical Coherence Tomography Scans: A Retrospective Multi-Centre Study

**DOI:** 10.3389/fmed.2022.860574

**Published:** 2022-06-15

**Authors:** An Ran Ran, Xi Wang, Poemen P. Chan, Noel C. Chan, Wilson Yip, Alvin L. Young, Mandy O. M. Wong, Hon-Wah Yung, Robert T. Chang, Suria S. Mannil, Yih Chung Tham, Ching-Yu Cheng, Hao Chen, Fei Li, Xiulan Zhang, Pheng-Ann Heng, Clement C. Tham, Carol Y. Cheung

**Affiliations:** ^1^Department of Ophthalmology and Visual Sciences, The Chinese University of Hong Kong, Hong Kong, Hong Kong SAR, China; ^2^Lam Kin Chung. Jet King-Shing Ho Glaucoma Treatment and Research Centre, The Chinese University of Hong Kong, Hong Kong, Hong Kong SAR, China; ^3^Department of Computer Science and Engineering, The Chinese University of Hong Kong, Hong Kong, Hong Kong SAR, China; ^4^Department of Radiation Oncology, Stanford University School of Medicine, Stanford, Palo Alto, CA, United States; ^5^Hong Kong Eye Hospital, Hong Kong, Hong Kong SAR, China; ^6^Department of Ophthalmology, Prince of Wales Hospital, Hong Kong, Hong Kong SAR, China; ^7^Department of Ophthalmology, Alice Ho Miu Ling Nethersole Hospital, Hong Kong, Hong Kong SAR, China; ^8^Tuen Mun Eye Centre, Hong Kong, Hong Kong SAR, China; ^9^Department of Ophthalmology, Byers Eye Institute, Stanford University, Palo Alto, CA, United States; ^10^Singapore Eye Research Institute, Singapore National Eye Centre, Singapore, Singapore; ^11^Duke-National University of Singapore Medical School, Singapore, Singapore; ^12^Department of Ophthalmology, Yong Loo Lin School of Medicine, National University of Singapore, Singapore, Singapore; ^13^Department of Computer Science and Engineering, The Hong Kong University of Science and Technology, Hong Kong, Hong Kong SAR, China; ^14^State Key Laboratory of Ophthalmology, Zhongshan Ophthalmic Centre, Sun Yat-sen University, Guangzhou, China

**Keywords:** artificial intelligence, deep learning, multi-task, glaucomatous optic neuropathy, glaucoma, myopic features, myopia, optical coherence tomography

## Abstract

**Purpose:**

We aim to develop a multi-task three-dimensional (3D) deep learning (DL) model to detect glaucomatous optic neuropathy (GON) and myopic features (MF) simultaneously from spectral-domain optical coherence tomography (SDOCT) volumetric scans.

**Methods:**

Each volumetric scan was labelled as GON according to the criteria of retinal nerve fibre layer (RNFL) thinning, with a structural defect that correlated in position with the visual field defect (i.e., reference standard). MF were graded by the SDOCT *en face* images, defined as presence of peripapillary atrophy (PPA), optic disc tilting, or fundus tessellation. The multi-task DL model was developed by ResNet with output of Yes/No GON and Yes/No MF. SDOCT scans were collected in a tertiary eye hospital (Hong Kong SAR, China) for training (80%), tuning (10%), and internal validation (10%). External testing was performed on five independent datasets from eye centres in Hong Kong, the United States, and Singapore, respectively. For GON detection, we compared the model to the average RNFL thickness measurement generated from the SDOCT device. To investigate whether MF can affect the model’s performance on GON detection, we conducted subgroup analyses in groups stratified by Yes/No MF. The area under the receiver operating characteristic curve (AUROC), sensitivity, specificity, and accuracy were reported.

**Results:**

A total of 8,151 SDOCT volumetric scans from 3,609 eyes were collected. For detecting GON, in the internal validation, the proposed 3D model had significantly higher AUROC (0.949 vs. 0.913, *p* < 0.001) than average RNFL thickness in discriminating GON from normal. In the external testing, the two approaches had comparable performance. In the subgroup analysis, the multi-task DL model performed significantly better in the group of “no MF” (0.883 vs. 0.965, *p*-value < 0.001) in one external testing dataset, but no significant difference in internal validation and other external testing datasets. The multi-task DL model’s performance to detect MF was also generalizable in all datasets, with the AUROC values ranging from 0.855 to 0.896.

**Conclusion:**

The proposed multi-task 3D DL model demonstrated high generalizability in all the datasets and the presence of MF did not affect the accuracy of GON detection generally.

## Introduction

Glaucoma is the leading cause of visual morbidity and blindness worldwide, and it is projected to affect 111.8 million people by 2040 (1, 2). Prompt and accurate detection of glaucoma is extremely important in preventing and reducing irreversible visual loss. Spectral-domain optical coherence tomography (SDOCT), a non-contact and non-invasive imaging technology for cross-sectional and three-dimensional (3D) view of the retina and optic nerve head (ONH), is now commonly used to evaluate glaucomatous optic neuropathy (GON), the structural change of glaucoma (3–5). SDOCT is widely used to quantify retinal nerve fibre layer (RNFL), neuro-retinal rim, and other inner retinal layers (e.g., ganglion cell layer, inner plexiform layer). SDOCT is sensitive and specific for detecting glaucoma, especially when combined with other ophthalmoscopic modalities (3, 4, 6).

Nevertheless, myopic features (MF), including peripapillary atrophy (PPA), optic disc tilting, and fundus tessellation, could influence GON identification based on RNFL thickness measurement alone, which should be considered when interpreting the optic disc and its circumpapillary regions for diagnosis (7). For example, PPA beta zone correlates with glaucoma, while gamma zone is related to axial globe elongation. A higher degree of vertical optic disc tilting is associated with a more temporally positioned RNFL thickness peak, and a higher degree of fundus tessellation is associated with thinner RNFL (8–10). Eyes with longer axial length are associated with significantly higher percentages of false-positive errors based on an SDOCT built-in normative database (11). Hence, evaluating GON using SDOCT based on RNFL thickness and built-in normative databases alone may not be reliable. As illustrated in Supplementary Figure 1A, MF can also result in thinning of RNFL thickness (i.e., outside of the normal RNFL range) in eyes without GON which is similar to eyes with GON (Supplementary Figure 1B). Other diagraphs and metrics, such as topographical ONH measurements, RNFL thickness map, RNFL deviation map, circumpapillary RNFL thickness with “double-hump pattern” should also be evaluated to differentiate these two pathologies carefully. For example, in purely myopic eyes, the “double-hump pattern” is still existed but with temporal shift due to optic disc tilting. The RNFL thickness map also shows normal thickness except that the angle between superior and inferior RNFL bundles is smaller. While in GON eyes, RNFL “double hump pattern” is altered and thinner RNFL thickness appears at specific regions. Thus, interpretation of the results requires experienced glaucoma specialists or highly trained assessors who have good knowledge on both GON and OCT limitations.

Deep learning (DL), composed of multiple processing layers, allows computational models to learn representative features with multiple levels of abstraction. These models showed promise in pattern recognition and image analysis (12). Currently, automated image analysis based on DL technology has been developed to detect GON from different kinds of images, such as fundus photographs (FP) and OCT images (13–20). We previously developed a 3D DL model to detect GON from SDOCT volumetric scans, which performed non-inferiorly to glaucoma specialists (21). However, all these DL algorithms only detected GON without learning features of Yes/No MF. Previous studies showed an increased risk of glaucoma among myopic eyes and high myopia is also a risk factor of GON progression (22, 23). Besides, there is a lack of knowledge whether MF affects DL model’s discriminative ability for GON detection. Having additional information on yes/no MF may help to evaluate subjects with glaucoma further. Multi-task learning is a training paradigm to train DL models with data from multiple tasks simultaneously, using shared representations to learn the common features between a collection of related tasks (24). It has been used in ultrasound, CT, and dermoscopic images (25–27), which showed potential advantages of integrating information across domains and extracting more general features for different tasks. Our previous work also applied multi-task technique for detecting GON and predicting visual field (VF) metrics (28), or detecting different kinds of retinal diseases (29, 30) from OCT images.

In this study, we aimed to train and validate a multi-task 3D DL model to analyze SDOCT volumetric scans and identify GON and MF simultaneously. We hypothesise that the model with multi-task technique would extract common features and achieve high generalizability for both tasks. Besides, the proposed model could achieve better or comparable performance when comparing with conventional RNFL thickness.

## Materials and Methods

This study was a multi-centred retrospective study. It was approved by the Research Ethics Committee of the Hospital Authority, Hong Kong (HK), the SingHealth Centralised Institutional Review Board, Singapore, and Stanford University’s Institutional Review Board/Ethics Committee, the United States (US). The study adhered to the Declaration of Helsinki. As the study involved only retrospective analysis using fully anonymized SDOCT images, informed consent was exempted.

### Training, Tuning, and Internal Validation Dataset

The dataset for training, tuning, and internal validation was inherited from our previous study (21). It was collected from existing database of electronic medical and research records at the Chinese University of Hong Kong (CUHK) Eye Centre and the Hong Kong Eye Hospital (HKEH). The inclusion criteria were (1) age equal to or older than 18 years old, (2) gradable SDOCT optic disc scans and *en face* images, (3) reliable VF tests, and (4) confirmed diagnosis of glaucoma or healthy subjects. The exclusion criteria were (1) other ocular or systemic diseases that may cause optic disc abnormalities or VF defect; (2) missing data on VF, SDOCT optic disc scans, or *en face* images. The non-glaucomatous subjects from the research centre were volunteers from existing cohorts in CUHK Eye Centre. The non-glaucomatous subjects from the eye clinics were subjects who seek for opportunistic eye check-ups. All study subjects underwent SDOCT imaging by Cirrus HD-OCT (Carl Zeiss Meditec, Inc., Dublin, CA, United States) using the optic disc cube scanning protocol, which generated the RNFL thickness map (6 mm^2^× 6 mm^2^) around the optic disc. The VF of each study subject was determined by static automated white-on-white threshold perimetry using the Humphrey Field Analyzer II (Carl Zeiss Meditec, Inc., Dublin, CA, United States). For each feasible eye, 3D SDOCT optic disc scan and 2D *en face* fundus image could be extracted simultaneously.

### External Testing Datasets

We used five independent datasets from other eye centres to test the performance of the DL model: External testing 1, Prince of Wales Hospital (PWH), HK; External testing 2, Tuen Mun Eye Centre (TMEC), HK; External testing 3, Alice Ho Miu Ling Nethersole Hospitals (AHNH), HK; External testing 4, Byers Eye Institute, Stanford University (Stanford), United States; and External testing 5, Singapore Eye Research Institute (SERI), Singapore. The inclusion criteria, exclusion criteria, VF and OCT device, and ground truth labelling for the external testing datasets were the same as the development dataset.

### Ground Truth Labelling

For the ground truth labelling, we first excluded ungradable images and then classified GON and MF in each gradable images, which was consistent with our previous studies (21, 31). The criteria were as follow:

Ungradable SDOCT images was defined as signal strength (SS) less than 5 or any artefacts influencing the measurement circle or > 25% of the peripheral area (31). GON was defined as RNFL thinning on gradable SDOCT images, with a structural defect that correlated in position with the VF defect which fulfilled the definition of glaucomatous VF defect (32). These eyes were labelled as “Yes GON.” Eyes without GON were defined as normal VF with no obvious glaucomatous optic disc cupping and loss of neuro-retinal rim, and these eyes were labelled as “No GON.”

Myopic features included presence of (1) PPA, chorioretinal thinning and disruptive of the retinal pigment epithelium (RPE) (33), (2) optic disc tilting, the ratio between the shortest and longest metres (tilt ratio) less than 0.8 (34), and (3) fundus tessellation, increased visibility of the large choroidal vessels outside of the parapapillary area (8). Eyes with one or more of these features were labelled as “Yes MF,” while eyes without any features were labelled as “No MF” (Supplementary Figure 2).

Following the definitions, SDOCT scans and LSO *en face* images were subjected to a tiered grading system by trained assessors, ophthalmologists, and glaucoma specialists, for assessing image quality, MF, and GON, respectively. Images were labelled when two graders arrived at the same categorization separately. For those cases on which the two graders did not arrive at the same categorization in their independent evaluations, the cases were reviewed and categorised by senior graders.

### Data Pre-processing

We applied standardisation and normalisation for data pre-processing. Specifically, standardisation was used to transfer data to have zero mean and unit variance, and normalisation rescaled the data to the range of 0–1. To alleviate the over-fitting issue, during the training process, we used several data augmentation techniques, including random cropping and random flipping at three axes, to enrich training samples for the 3D SDOCT volumetric data. Consequently, the final input size of the network was 200 × 1000 × 200.

We implemented the DL model using *Keras* package and python on a workstation equipped with 3.5 GHz Intel^®^ Core™ i7-5930K CPU and GPUs of Nvidia GeForce GTX Titan X. We set the learning rate as 0.0001 and optimised the weights of the networks with Adam stochastic gradient descent algorithm.

### Development of the Multi-Task Deep Learning Model

**FIGURE 1:**
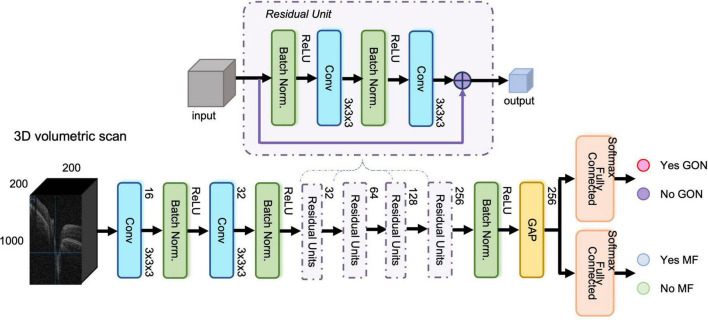
The structure of the three-dimensional (3D) multi-task deep learning model. The proposed network included three modules, (1) shared feature extraction module, (2) glaucomatous optic neuropathy (GON) classification module, and (3) myopic features (MF) detection module, respectively. It was built based on ResNet-37 network with 3D convolutional layers and global average pooling layer. The input was an OCT volumetric scan of size 200 × 1000 × 200 pixels after image pre-processing and the output was Yes/No GON and Yes/No MF.

The proposed network included three modules, (1) shared feature extraction module, (2) GON classification module, and (3) MF detection module, respectively. The constructed network was similar to our previous study (21) with ResNet-37 as the backbone. We used shortcut connections to perform identity mapping and evade vanishing gradient problem during backpropagation. We removed the fully connected layer from the 3D ResNet-37. This module acted as the shared feature extraction module. In the GON classification module, a fully connected layer with softmax activation accepted the feature from the first module and output the classification probabilities for “Yes GON” and “No GON.” Likewise, there was also a fully connected layer with softmax activation in the MF detection module. Figure 1 displays the structure of the multi-task 3D DL model.

All gradable SDOCT volumetric scans collected from CUHK Eye Center and HKEH were randomly divided for training (80%), tuning (10%), and internal validation (10%) at the patient level. In each set, the ratio of “Yes GON and Yes MF,” “Yes GON and No MF,” “No GON and Yes MF,” and “No GON and No MF” was similar, and multiple images from the same subjects were in the same set to prevent leakage or performance overestimation. We trained the multi-task DL model from scratch, and the tuning dataset was used to select and modify the optimum model during training. During the training, tuning, and internal validation, we observed the training-validation curve to evaluate over-fitting issue, which could also provide a further reference to the generalizability of the models. Additionally, SDOCT volumetric scans from PWH, TMEC, AHNH, Stanford, and SERI were used for external testing.

Finally, we generated heatmaps for selected eyes by class activation map (CAM) (35) to evaluate the model performance qualitatively.

### Development of Single-Task Models and a 2D Model for Performance Comparison

We trained and tested two 3D single-task DL models using the same split dataset as the proposed 3D multi-task model for GON and MF detection, respectively. We also used segmentation-free 2D OCT B-scans as the input to train and test a 2D multi-task DL model using the same split dataset as the proposed 3D multi-task model. Each OCT volumetric scan contained 200 B-scans and the mean predictions of these B-scans were used as the volume-level prediction. All the models were tested on the same testing sets for final performance comparison.

### Statistical Analysis

The statistical analyses were performed by RStudio Version 1.1.463 (2009–2018 RStudio, Inc.). One-way ANOVA and chi-square test were performed for numerical and categorical data, respectively, to analyse demographic characteristics of all the participants and data variances of different datasets. The area under the receiver operating characteristic curve (AUROC) with 95% confidence interval (CI), sensitivity, specificity, and accuracy were calculated to evaluate the discriminative performance (Yes/No GON and Yes/No MF) of the 3D multi-task DL model, 3D single-task model, and 2D multi-task DL model in all the datasets. For GON detection, we further compared the performance of the proposed multi-task 3D DL model to that of the average RNFL thickness measurement generated from the SDOCT device. Delong test was used to compare the AUROCs of different methods. To investigate whether MF can affect the model’s performance on GON detection, we also conducted subgroup analyses in groups stratified by Yes/No MF. All the hypotheses tested were two-sided, and a *p*-value of less than 0.05 were considered statistically significant.

## Results

Table 1 displays the characteristics of all the datasets. We investigated 2,541 subjects, including 1,452 glaucoma patients and 1,089 non-glaucomatous subjects. In the total of 8,151 SDOCT volumetric scans from 3,609 eyes were collected. To develop the multi-task DL model, we used 3,919 scans from 1,196 eyes for training, 460 scans from 180 eyes for tuning, and 454 scans from 196 eyes for internal validation. For the external testing, we collected five independent datasets from different eye centres and used 545, 267, 515, 933, and 1,058 SDOCT volumetric scans, from 417, 261, 428, 399, and 532 eyes, respectively. There was no significant difference in age, gender (male vs. female), and eye (right vs. left) of subjects among all the groups in tuning and internal validation set. For training set, age and gender had a significant difference.

**TABLE 1:** Summary of the study subjects and data distribution.

	Total	yes GON and yes MF	no GON and yes MF	yes GON and no MF	no GON and no MF	*p*-value
**Training dataset**						
No. of SDOCT scans	3,919	1,679	890	629	721	\
No. of patients	951	390	216	166	179	\
Gender (male/female)	402/549	183/207	83/133	72/94	64/115	**0.047**
Age, years (mean ± SD)	65.6 ± 12.6	67.3 ± 12.9	64.7 ± 12.3	66.6 ± 11.7	62.4 ± 12.2	**< 0.001**
No. of eyes	1,196	495	280	192	229	\
Eye (right/left)	579/617	242/253	142/138	90/102	105/124	0.70
**Tuning dataset**						
No. of SDOCT scans	460	195	163	32	70	\
No. of patients	132	56	43	13	20	\
Gender (male/female)	52/80	22/34	16/27	8/5	6/14	0.32
Age, years (mean ± SD)	64.7 ± 12.7	66.3 ± 13.2	64.0 ± 13.0	63.8 ± 13.0	66.6 ± 10.3	0.66
No. of eyes	180	83	51	17	29	\
Eye (right/left)	89/91	39/44	27/24	8/9	15/14	0.91
**Internal validation dataset**						
No. of SDOCT scans	454	205	114	36	99	\
No. of patients	143	63	38	19	23	\
Gender (male/female)	71/72	32/31	17/21	9/10	13/10	0.83
Age, years (mean ± SD)	67.0 ± 12.2	68.9 ± 12.6	63.3 ± 13.4	70.6 ± 11.1	65.1 ± 7.7	0.06
No. of eyes	196	96	44	22	34	\
Eye (right/left)	103/93	51/45	25/19	9/13	18/16	0.68
**External testing dataset 1**						
No. of SDOCT scans	545	215	125	79	126	\
No. of patients	307	114	79	47	67	\
Gender (male/female)	133/174	55/59	35/44	19/28	24/43	0.42
Age, years (mean ± SD)	68.6 ± 11.6	70.4 ± 11.8	67.3 ± 11.6	72.6 ± 10.6	64.2 ± 10.4	**< 0.001**
No. of eyes	417	156	104	63	94	\
Eye (right/left)	211/206	80/76	55/49	28/35	48/46	0.75
**External testing dataset 2**						
No. of SDOCT scans	267	74	61	69	63	\
No. of patients	193	52	48	51	42	\
Gender (male/female)	97/96	30/22	23/25	23/28	21/21	0.62
Age, years (mean ± SD)	60.5 ± 12.4	66.6 ± 7.1	55.6 ± 16.8	62.0 ± 9.6	56.5 ± 11.3	**< 0.001**
No. of eyes	261	70	61	69	61	\
Eye (right/left)	137/124	39/31	33/28	31/38	34/27	0.53
**External testing dataset 3**						
No. of SDOCT scans	515	296	101	66	52	\
No. of patients	294	156	66	41	31	\
Gender (male/female)	168/126	95/61	37/29	22/19	14/17	0.40
Age, years (mean ± SD)	62.8 ± 12.5	64.2 ± 10.9	60.4 ± 14.0	65.9 ± 10.8	57.2 ± 16.4	**0.004**
No. of eyes	428	243	86	54	45	\
Eye (right/left)	213/215	121/122	44/42	26/28	22/23	0.99
**External testing dataset 4**						
No. of SDOCT scans	933	477	57	241	158	\
No. of patients	249	108	19	58	64	\
Gender (male/female)	109/136	53/54	11/8	22/35	23/39	0.40
Age, years (mean ± SD)	66.0 ± 14.9	70.2 ± 13.6	64.5 ± 11.8	67.0 ± 16.6	58.5 ± 13.3	**< 0.001**
No. of eyes	399	187	25	92	95	\
Eye (right/left)	205/194	96/91	13/12	48/44	48/47	0.99
**External testing dataset 5**						
No. of SDOCT scans	1058	287	171	222	378	\
No. of patients	272	65	56	53	98	\
Gender (male/female)	155/117	43/22	32/24	35/18	45/53	**0.02**
Age, years (mean ± SD)	68.6 ± 8.5	67.5 ± 8.9	70.6 ± 8.4	69.2 ± 8.3	67.9 ± 8.4	0.17
No. of eyes	532	126	88	119	199	\
Eye (right/left)	263/269	61/65	56/32	52/67	94/105	0.80

*GON, glaucomatous optic neuropathy; MF, myopic features.*

*Training, Tuning, and Internal validation: CUHK Eye Centre and Hong Kong Eye Hospital (HKEH), Hong Kong SAR.*

*External testing dataset 1: Prince of Wales Hospital (PWH), Hong Kong SAR.*

*External testing dataset 2: Tuen Mun Eye Centre (TMEC), Hong Kong SAR.*

*External testing dataset 3: Alice Ho Miu Ling Nethersole Hospital (AHNH), Hong Kong SAR.*

*External testing dataset 4: Byers Eye Institute, Stanford University (Stanford), the United States.*

*External testing dataset 5: Singapore Eye Research Institute (SERI), Singapore.*

*One-way ANOVA and χ^2^ test were used for numerical and categorical data, respectively for comparison between “yes GON and yes MF,” “yes GON and no MF,” “no GON and yes MF,” and “no GON and no MF” groups. p-values in bold were AUROC values with significant difference.*

**FIGURE 2:**
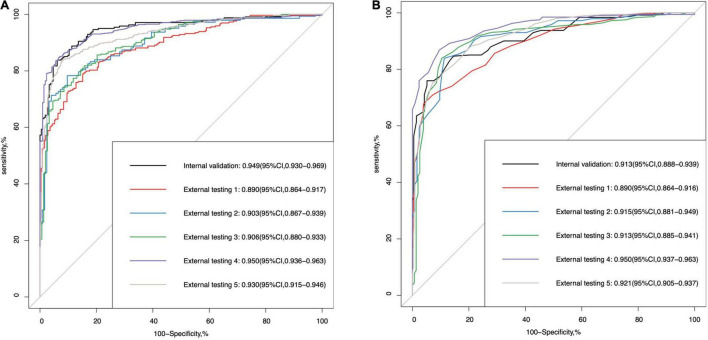
The area under the receiver operating characteristic (ROC) curve (AUROC) values of **(A)** the multi-task deep learning model and **(B)** retinal nerve fibre layer thickness in internal validation and external testing for glaucomatous optic neuropathy detection.

Table 2 demonstrates the performance of the 3D multi-task DL model for GON identification and the comparison to average RNFL thickness, a 3D single-task DL model, and a 2D multi-task model. In the internal validation dataset, the proposed model had significantly higher AUROC (0.949 vs. 0.913, *p* < 0.001) than that of RNFL thickness. In the five external testing datasets, the two methods (DL model vs. RNFL thickness) had comparable AUROC values (0.890 vs. 0.890, 0.903 vs. 0.915, 0.906 vs. 0.913, 0.950 vs. 0.950, and 0.930 vs. 0.921, respectively). Figures 2A,B shows the ROC curves and AUROC values using the 3D multi-task DL model and RNFL thickness to identify GON in internal validation and external testing. The proposed 3D multi-task model also achieved generally better performance than a 3D single-task model and a 2D multi-task model.

**TABLE 2:** The discriminative performance of the multi-task 3D deep learning model for detecting glaucomatous optic neuropathy and the comparison to average retinal nerve fibre layer thickness, a single-task 3D deep learning model, and a multi-task 2D deep learning model in all datasets.

	AUROC (95% CI)	*p*-value	Sensitivity, % (95% CI)	Specificity, % (95% CI)	Accuracy, % (95% CI)	PPV, % (95% CI)	NPV, % (95% CI)
**Internal validation**							
3D multi-task DL	0.949 (0.930–0.969)	**\**	88.0 (80.9–95.9)	91.6 (81.7–97.2)	89.4 (86.6–92.1)	92.2 (85.5–97.0)	87.1 (81.3–94.7)
Average RNFL thickness	0.913 (0.888–0.939)	**< 0.001**	80.1 (72.2–88.4)	92.5 (84.0–97.2)	85.5 (82.2–88.6)	92.0 (85.7–96.9)	80.5 (75.0–86.7)
3D single-task DL	0.941 (0.920–0.961)	0.53	86.3 (73.4–95.0)	88.3 (78.4–98.6)	87.0 (84.1–90.1)	89.4 (82.8–98.4)	85.2 (76.4–93.5)
2D multi-task DL	0.940 (0.919–0.961)	0.53	84.7 (78.4–92.1)	92.5 (84.0–97.2)	88.3 (85.5–91.0)	92.6 (86.2–97.0)	84.4 (79.4–90.7)
**External testing 1**							
3D multi-task DL	0.890 (0.864–0.917)	\	78.9 (70.4–86.4)	86.1 (77.3–92.8)	82.0 (78.7–85.1)	86.9 (81.4–92.4)	77.7 (72.1–83.3)
Average RNFL thickness	0.890 (0.864–0.916)	0.96	69.9 (63.7–76.7)	94.8 (89.2–98.0)	81.2 (78.3–84.2)	94.0 (88.7–97.5)	73.0 (69.5–77.3)
3D single-task DL	0.893 (0.867–0.919)	0.88	82.3 (70.1–89.8)	81.7 (72.9–92.0)	81.8 (78.7–85.0)	84.2 (79.2–91.6)	79.7. (72.0–86.2)
2D multi-task DL	0.900 (0.876–0.925)	0.58	82.7 (75.5–91.8)	82.1 (70.5–88.8)	82.3 (78.9–85.3)	84.3 (78.4–89.2)	80.2 (74.7–88.2)
**External testing 2**							
3D multi-task DL	0.903 (0.867–0.939)	\	77.6 (67.1–86.7)	91.9 (83.1–98.4)	84.3 (80.2–88.4)	92.1 (85.0–98.2)	78.4 (72.0–85.4)
Average RNFL thickness	0.915 (0.881–0.949)	0.38	85.3 (78.3–93.7)	88.7 (77.4–93.6)	86.5 (82.4–90.3)	89.4 (82.5–94.1)	83.7 (77.9–91.6)
3D single-task DL	0.883 (0.841–0.925)	0.48	83.9 (69.2–93.7)	83.1 (70.2–94.4)	83.2 (79.0–87.3)	85.2 (78.1–94.1)	81.8. (72.1–90.8)
2D multi-task DL	0.882 (0.843–0.922)	0.45	81.1 (67.1–89.5)	83.1 (73.4–93.6)	82.0 (77.5–86.2)	84.9 (78.5–92.8)	79.3 (70.8–86.8)
**External testing 3**							
3D multi-task DL	0.906 (0.880–0.933)	\	79.7 (68.5–88.1)	88.9 (79.1–96.7)	82.1 (76.5–86.6)	94.4 (90.5–98.2)	64.9 (56.2–74.7)
Average RNFL thickness	0.913 (0.885–0.941)	0.53	84.8 (80.4–90.9)	88.9 (81.1–94.1)	86.2 (82.9–89.3)	94.8 (91.7–97.1)	71.4 (65.6–79.3)
3D single-task DL	0.898 (0.868–0.928)	0.70	87.1 (78.0–92.5)	79.9 (70.5–88.5)	84.5 (79.4–88.2)	90.6 (87.4–94.3)	72.8 (62.2–81.4)
2D multi-task DL	0.903 (0.876–0.931)	0.89	82.4 (68.2–89.0)	84.9 (76.3–95.7)	82.9 (76.4–86.9)	92.7 (89.2–97.4)	67.6 (56.6–76.4)
**External testing 4**							
3D multi-task DL	0.950 (0.936–0.963)	\	85.2 (79.0–92.5)	94.0 (86.5–98.1)	87.3 (83.2–91.1)	97.9 (95.8–99.3)	65.6 (58.1–77.4)
Average RNFL thickness	0.950 (0.937–0.963)	0.85	87.0 (79.5–90.5)	90.6 (85.9–96.2)	87.9 (83.5–90.3)	96.9 (95.5–98.7)	67.5 (58.4–73.5)
3D single-task DL	0.929 (0.911–0.947)	0.08	83.3 (74.1–92.9)	87.4 (77.2–95.4)	84.5 (78.8–89.6)	95.7 (93.0–98.1)	61.2 (52.3–76.8)
2D multi-task DL	0.939 (0.923–0.955)	0.31	88.0 (76.3–93.2)	84.7 (77.7–94.9)	87.1 (80.1–90.4)	95.1 (93.2–98.0)	67.8 (54.0–77.8)
**External testing 5**							
3D multi-task DL	0.930 (0.915–0.946)	\	83.9 (80.4–87.2)	92.2 (89.6–94.7)	88.2 (86.3–90.0)	90.9 (88.2–93.6)	86.1 (83.6–88.6)
Average RNFL thickness	0.921 (0.905–0.937)	0.15	80.2 (73.1–90.2)	89.1 (78.1–94.5)	84.5 (82.5–86.6)	87.2 (79.1–92.9)	83.1 (78.9–89.7)
3D single-task DL	0.936 (0.922–0.951)	0.31	84.1 (80.8–88.8)	92.4 (87.1–94.9)	88.3 (86.3–90.2)	91.1 (86.2–93.8)	86.2 (83.8–89.5)
2D multi-task DL	0.938 (0.924–0.953)	0.45	84.1 (80.2–88.2)	93.8 (89.6–96.4)	89.0 (87.2–90.8)	92.5 (88.6–95.4)	86.4 (83.7–89.4)

*AUROC, area under the receiver operator characteristic curve; CI, confidence interval; PPV, positive predictive value; NPV, negative predictive value; DL, deep learning; RNFL, retinal nerve fibre layer. p-values in bold were AUROC values with significant difference.*

In the sub-analysis stratified by Yes/No MF, there was no significant difference in internal validation (0.938 vs. 0.952, *p*-value = 0.65) and External testing 1 (0.884 vs. 0.896, *p*-value = 0.67), External testing 2 (0.926 vs. 0.900, *p*-value = 0.47), External testing 4 (0.941 vs. 0.940, *p*-value = 0.95), and External testing 5 (0.923 vs. 0.925, *p*-value = 0.93). In External testing 3, the multi-task DL model performed significantly better in the group of “no MF” (0.883 vs. 0.965, *p*-value < 0.001) (Table 3).

**TABLE 3:** Subgroup analysis for detecting glaucomatous optic neuropathy in groups stratified by yes/no myopic features.

	AUROC (95% CI)	*p-value	Sensitivity, % (95% CI)	Specificity, % (95% CI)	Accuracy, % (95% CI)	PPV, % (95% CI)	NPV, % (95% CI)
**Internal validation**							
yes MF	0.938 (0.913–0.962)	0.65	85.4 (77.6–93.7)	90.4 (79.8–95.6)	87.2 (82.8–90.6)	94.0 (89.3–97.2)	77.3 (69.9–87.6)
no MF	0.952 (0.896–1)	\	94.4 (83.3–100)	95.0 (87.9–100)	94.1 (88.9–97.8)	86.8 (73.9–100)	97.8 (94.1–100)
**External testing 1**							
yes MF	0.884 (0.849–0.918)	0.67	78.1 (67.0–85.6)	88.8 (80.8–96.8)	81.8 (76.8–86.2)	92.1 (87.8–97.2)	70.1 (62.6–77.6)
no MF	0.896 (0.851–0.941)	\	83.5 (68.4–93.7)	83.3 (72.2–94.4)	83.4 (77.6–88.3)	75.8 (66.4–89.6)	88.8 (82.4–94.9)
**External testing 2**							
yes MF	0.926 (0.879–0.972)	0.47	87.8 (75.7–97.3)	90.2 (78.7–98.4)	88.9 (83.7–93.3)	91.8 (84.3–98.5)	86.6 (76.3–96.2)
no MF	0.900 (0.849–0.951)	\	79.7 (62.3–95.7)	90.5 (68.3–100)	83.3 (78.0–88.6)	90.0 (75.9–100)	80.3 (70.0–94.8)
**External testing 3**							
yes MF	0.883 (0.847–0.919)	**< 0.001**	74.7 (64.9–86.8)	90.1 (76.2–97.0)	78.6 (72.3–85.4)	95.5 (91.2–98.6)	54.8 (47.7–67.5)
no MF	0.965 (0.937–0.992)	\	90.9 (78.8–98.5)	94.2 (82.7–100)	91.5 (86.4–95.8)	94.7 (87.5–100)	89.3 (78.1–98.0)
**External testing 4**							
yes MF	0.941 (0.916–0.966)	0.95	85.7 (79.9–91.2)	94.7 (87.7–100)	86.7 (81.7–91.2)	99.3 (98.3–100)	44.4 (36.3–55.4)
no MF	0.940 (0.918–0.962)	\	84.7 (77.2–90.9)	91.8 (85.4–97.5)	87.5 (84.0–90.7)	94.1 (90.2–97.9)	79.8 (73.1–86.4)
**External testing 5**							
yes MF	0.923 (0.897–0.949)	0.93	87.8 (78.1–92.3)	88.3 (81.3–95.3)	87.6 (83.4–90.6)	92.5 (88.9–96.6)	80.8 (71.5–87.1)
no MF	0.925 (0.902–0.947)	\	78.8 (73.0–84.2)	95.5 (92.6–97.6)	89.2 (86.7–91.5)	91.2 (86.0–95.2)	88.4 (85.8–91.0)

*Z-test was used to compare the AUROC values between yes/no myopic features groups. p-values in bold were AUROC values with significant difference.

Table 4 and Supplementary Figure 3 illustrate the DL model’s performance to detect MF. It was generalizable in internal validation and five external testing datasets, with the AUROC values of 0.892 (95% CI, 0.860–0.924), 0.885 (95% CI, 0.855–0.915), 0.855 (95% CI, 0.811–0.899), 0.886 (95% CI, 0.856–0.916), 0.866 (95% CI, 0.843–0.888), and 0.875 (95% CI, 0.854–0.896), respectively. When comparing with a 3D single-task model and a 2D multi-task model, the proposed 3D multi-task showed better performance with higher generalizability in external testing.

**TABLE 4:** The discriminative performance of the multi-task 3D deep learning model for detecting myopic feature and the comparison to a single-task 3D deep learning model in all datasets.

	AUROC (95% CI)	*p*-value	Sensitivity, % (95% CI)	Specificity, % (95% CI)	Accuracy, % (95% CI)	PPV, % (95% CI)	NPV, % (95% CI)
**Internal validation**							
3D multi-task DL	0.892 (0.860–0.924)	\	79.6 (71.8–92.2)	86.7 (72.6–94.1)	81.9 (77.1–87.4)	93.3 (88.6–96.7)	64.6 (57.5–80.2)
3D single-task DL	0.873 (0.839–0.906)	0.39	79.6 (74.0–88.1)	83.7 (73.3–89.6)	80.8 (76.9–84.8)	91.8 (88.2–94.7)	63.4. (57.7–72.6)
2D multi-task DL	0.861 (0.823–0.900)	0.22	81.4 (74.9–88.4)	83.7 (74.8–90.4)	81.9 (78.0–85.9)	92.1 (88.9–94.9)	65.5 (59.0–73.9)
**External testing 1**							
3D multi-task DL	0.885 (0.855–0.915)	\	83.8 (74.4–93.8)	81.5 (69.3–90.2)	83.1 (79.1–86.6)	88.2 (83.2–92.9)	75.5 (67.3–87.5)
3D single-task DL	0.851 (0.818–0.884)	0.13	81.5 (67.7–87.4)	78.1 (70.7–89.8)	80.0 (75.1–83.3)	86.0. (82.6–91.6)	71.6 (62.1–78.0)
2D multi-task DL	0.818 (0.781–0.855)	**0.006**	83.5 (73.8–92.9)	67.3 (54.6–76.6)	77.3 (72.8–80.9)	80.7 (76.8–84.9)	71.0 (62.6–83.1)
**External testing 2**							
3D multi-task DL	0.855 (0.811–0.899)	\	83.7 (68.9–91.9)	76.5 (66.7–87.9)	79.8 (74.9–84.6)	78.3 (72.5–86.5)	81.8 (72.9–89.5)
3D single-task DL	0.806 (0.755–0.858)	0.16	65.2 (45.9–83.0)	86.4 (67.4–99.2)	74.9 (70.4–79.4)	82.8 (71.2–98.4)	70.8 (63.7–80.3)
2D multi-task DL	0.799 (0.747–0.852)	0.12	69.6 (54.1–87.4)	81.1 (60.6–92.4)	74.9 (70.0–79.4)	78.5 (68.1–89.0)	72.3 (65.6–83.3)
**External testing 3**							
3D multi-task DL	0.886 (0.856–0.916)	\	78.3 (72.8–84.6)	88.1 (79.7–94.1)	80.6 (76.3–84.7)	95.6 (93.0–97.8)	54.7 (49.1–61.9)
3D single-task DL	0.831 (0.795–0.868)	**0.03**	65.8 (60.4–72.4)	95.3 (89.6–99.1)	72.7 (68.5–77.0)	97.9 (95.6–99.6)	45.7 (42.1–50.3)
2D multi-task DL	0.850 (0.812–0.887)	0.15	72.4 (64.1–81.2)	88.7 (78.3–95.3)	76.2 (70.5–81.4)	95.4 (92.1–97.9)	49.2 (43.4–56.8)
**External testing 4**							
3D multi-task DL	0.866 (0.843–0.888)	\	68.4 (62.9–77.0)	95.0 (86.2–97.7)	79.5 (77.1–82.0)	94.7 (88.0–97.6)	69.1 (66.0–74.0)
3D single-task DL	0.849 (0.825–0.873)	0.34	70.6 (62.6–79.8)	84.7 (74.9–91.2)	76.5 (73.6–79.2)	85.9 (80.6–90.7)	68.3 (64.1–74.0)
2D multi-task DL	0.859 (0.836–0.883)	0.70	72.8 (66.1–86.2)	85.5 (70.9–90.7)	78.2 (75.5–81.1)	86.8 (79.7–90.7)	70.2 (66.3–79.7)
**External testing 5**							
3D multi-task DL	0.875 (0.854–0.896)	\	84.1 (70.7–90.2)	76.5 (69.3–88.3)	79.8 (77.0–82.2)	73.2 (68.7–82.4)	86.2 (79.6–90.4)
3D single-task DL	0.886 (0.866–0.907)	0.18	83.2 (78.0–88.9)	83.8 (77.5–88.3)	83.4 (80.9–85.6)	79.6 (74.6–84.1)	86.7 (83.7–90.3)
2D multi-task DL	0.862 (0.840–0.884)	0.39	85.2 (69.0–90.4)	72.3 (65.5–87.2)	78.1 (75.4–80.7)	70.3 (66.3–80.9)	86.3 (78.4–90.3)

*p-values in bold were AUROC values with significant difference.*

The training-tuning curve (Supplementary Figure 4) showed that the multi-task 3D DL model converged approximately around the 30th epoch and kept stable without significant oscillation after the 50th epoch. This finding, combined with the discriminative performance results in all the datasets, suggested that the multi-task 3D DL model had learned general knowledge to identify both GON and MF, and was not overfitted.

**FIGURE 3:**
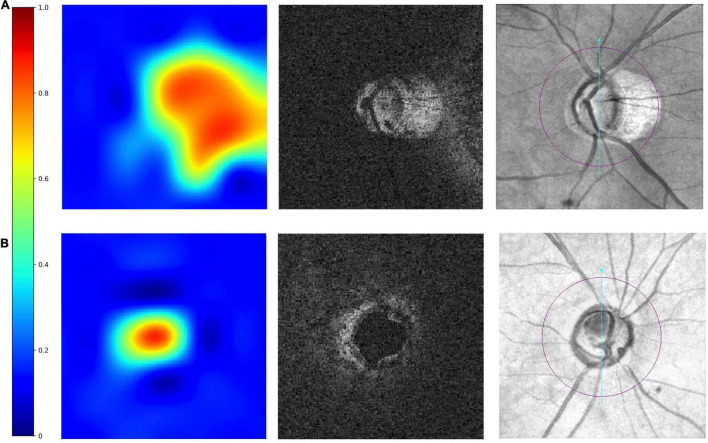
Examples of truly detected eyes by the multi-task 3D DL model. From left to right were heatmaps, raw images, and the corresponding *en face* fundus images. The red–orange-coloured area on heatmaps has the most discriminatory power to detect MF. The heatmaps shows in panel **(A)** an eye with myopic features (peripapillary atrophy, PPA), the optic disc area and the areas with PPA is red-orange-coloured. In panel **(B)** an eye without myopic features, only optic disc was red-orange-coloured.

On the heatmaps (Figure 3), the red-orange-coloured area has the most discriminatory power to detect MF. The optic disc and PPA areas were red-orange-coloured in the truly detected eye with PPA. It demonstrated that the 3D multi-task DL model could discriminate MF around the ONH. While for the truly detected SDOCT scans as “no MF,” the heatmaps showed that only the optic disc area was red-orange-coloured.

## Discussion

To the best of our knowledge, the proposed multi-task 3D DL model is the first reported attempt to automatically detect GON and MF from SDOCT volumetric data simultaneously. The results showed a generalised performance for both tasks among the datasets and the presence of MF did not significantly affect DL model’s ability to identify GON.

Compared with a single task model, a multi-task model learned shared features from multiple tasks simultaneously. These shared features can potentially increase data efficiency and yield faster learning speed for related or down-stream tasks, which may alleviate DL’s weakness of requiring large-scale data and computation power (24). The proposed 3D DL multi-task model showed higher generalizability to detect GON when comparing with our previous single task model (21). When tested on two newly collected unseen datasets from HK (External testing 3) and Singapore (External testing 5), it achieved AUROC values of 0.906 and 0.930, respectively. To be more precise, we also trained single-task models with the exact same split data as the multi-task model. We found that the multi-task model had generally better performance for GON detection. The sub-analysis also reflected that except External testing 3, the presence or absence of MF did not influence the discriminative performance of the proposed multi-task DL model for GON detection in internal validation and external testing, which proved that during the training process, the multi-task model learned effective features to identify GON in eyes with or without MF, so that when testing on unseen datasets, the performance of GON detection will not be influenced by presence or absence of MF. Hence, after applying the multi-task learning strategy and providing additional information of Yes/No MF during the training process, the proposed multi-task 3D DL model learned more general features and demonstrated a robust discriminative ability in the task of identifying GON when tested on different datasets.

Compared with conventional method, i.e., RNFL thickness measurement, the DL model performed better in internal validation and comparable in five external testing datasets for GON identification. In addition to the good performance, this automated DL model can provide a straightforward classification result, i.e., Yes or No GON, which was more advantageous than current discriminative capability based on RNFL thickness and built-in normative databases as the RNFL thickness is affected by various factors including axial length, myopia, age, optic disc size, and SS (36–41). Experienced ophthalmologists are needed to interpret and classify GON based on a series of outputs from the SDOCT report. Therefore, the proposed multi-task model can potentially be applied in primary settings without glaucoma specialists or even ophthalmologists on site, which may help primary care clinicians to identify GON simply from the binary output of the DL model, and then refer to ophthalmologists.

In addition, the multi-task DL model also offered an output of “Yes/No MF” for each image with good and consistent performance in internal and external datasets. Myopia is one of the risk factors for glaucoma (23, 42–44) and the ONH deformations in myopic eyes may predispose toward glaucoma (45). Features observed on areas around ONH on the fundus, such as the location of principal RNFL bundles, optic disc tilting, and optic disc torsion, were also related to spherical error and glaucoma severity (7, 46). Our multi-task 3D DL model can detect the presence or absence of these features and give clinicians more information. The heatmaps also proved that for MF discrimination, the 3D multi-task DL model paid more attention on the ONH and the surrounding areas. Besides, the multi-task model showed significantly higher generalizability when comparing with single-task model for MF detection. Thus, it further proved aforementioned advantage of the multi-task model that learning both GON and MF features during training could potentially improve the model’s generalizability for both tasks when testing on unseen datasets.

Our study has several strengths. First, we collected our datasets from different eye centres from different countries and regions including different ethnic backgrounds. It performed consistently well in all the datasets. The training-tuning curves also illustrated that the proposed DL model was not overfitted. Thus, our multi-task 3D DL model could potentially be applied on other unseen datasets, even among different populations. Second, we used multi-task technique to provide additional information on MF without influencing GON identification. Current model could detect two kinds of abnormalities simultaneously and will potentially assist clinicians with less experience in classifying glaucoma. Third, the multi-task DL model can generate straightforward output of Yes/No GON and MF and can be incorporated with our previously developed image quality assessment model in the future (31), which will further strengthen SDOCT as a screening tool in settings without sufficient ophthalmologists on site. Fourth, we developed a 3D multi-task model which could use of all the information in the volumetric scan and showed generally better performance than 2D model trained with B-scans for both tasks. It will provide volume-level output which would be more straightforward for physicians (non-ophthalmologists) and could also save manpower or computation power to deal with large number of B-scans. One of the limitations was that we used SDOCT paired *en face* photographs instead of colour FP to label MF due to the lack of unpaired OCT and FP in most patients. Besides, we only labelled the Yes/No MF instead of Yes/No myopia as the data of spherical error and AL for most of the patients was lacking due to the nature of retrospective study. However, information of anatomical features could be more useful when detecting GON in myopic eyes. In future, we may obtain a small dataset with paired OCT, FP, spherical error, or AL, for data annotation and apply advanced DL techniques, such as generative adversarial network (GAN) (47) or semi-supervised learning (28), to generate pseudo-labels for other data, which will further refine our model and enhance its feasibility to detect GON in high myopic eyes. We also intend to investigate whether increase the number of related tasks will further enhance the DL model’s discriminative ability and data-efficiency for GON detection.

In conclusion, with multi-task learning technique, the proposed 3D DL model demonstrated high generalizability in all the datasets to detect GON and MF simultaneously. It would potentially enhance clinicians’ capability to identify GON in eyes with MF and be applied in primary settings without sufficient specialists on site.

## Data Availability Statement

The de-identified individual participant data, the study protocol, the statistical analysis plan, and the coding are available upon reasonable request. Such requests are decided on a case-by-case basis. Proposals should be directed to CYC, carolcheung@cuhk.edu.hk.

## Ethics Statement

The studies involving human participants were reviewed and approved by the Research Ethics Committee of the Hospital Authority, Hong Kong; the SingHealth Centralized Institutional Review Board, Singapore; Stanford University’s Institutional Review Board/Ethics Committee, the United States. The patients/participants provided their written informed consent to participate in this study.

## Author Contributions

CYC and AR conceived and designed the study and wrote the initial draft. XW developed and validated the DL model supervised by HC and P-AH with the help of clinical input from AR, CYC, PC, NC, MW, and CT. PC, MW, and CT provided data from CUHK Eye Centre and HKEH. NC, WY, and AY provided data from PWH and AHNH. H-WY provided the data from TMEC. SM and RC provided the data from the Stanford. YT and C-YC provided the data from SERI. AR and XW performed the statistical analysis. XZ and FL provided clinical support during the model development. All authors subsequently critically edited the report and read and approved the final report.

## Conflict of Interest

The authors declare that the research was conducted in the absence of any commercial or financial relationships that could be construed as a potential conflict of interest.

## Publisher’s Note

All claims expressed in this article are solely those of the authors and do not necessarily represent those of their affiliated organizations, or those of the publisher, the editors and the reviewers. Any product that may be evaluated in this article, or claim that may be made by its manufacturer, is not guaranteed or endorsed by the publisher.
