# Supplementary material for: Three-Dimensional Multi-Task Deep Learning Model to Detect Glaucomatous Optic Neuropathy and Myopic Features From Optical Coherence Tomography Scans: A Retrospective Multi-Centre Study
Source: Front Med (Lausanne). 2022 Jun 15;9:860574. doi: 10.3389/fmed.2022.860574 (PMC9240220; doi:10.3389/fmed.2022.860574)

Supplementary Material

## Supplementary Figures

**Supplementary Figure 1.** Examples of a myopic patient without glaucoma (A) and a myopic patient with glaucoma patient (B). Myopic features can also result in thinner RNFL thickness outside the normal RNFL range in eyes without GON which is similar to eyes with glaucomatous optic neuropathy (red box). Other diagraphs and metrics, such as topographical optic nerve head measurements, RNFL thickness map, RNFL deviation map, RNFL circular thickness with double hump patterns should also be evaluated to differentiate these two pathologies. For example, in the purely myopic eyes, the double hump patterns are still existed but with temporal shift due to optic disc tilting (blue arrow). The RNFL thickness map also shows normal thickness except that the angle between superior and inferior RNFL bundles is smaller (red arrow). While in GON eyes, there are no double hump patterns (blue arrow) and the RNFL thickness map shows inferior and superior thinning (red arrow). Thus, interpretation of the results requires experienced glaucoma specialists or highly trained assessors who have good knowledge of OCT limitations.

A.


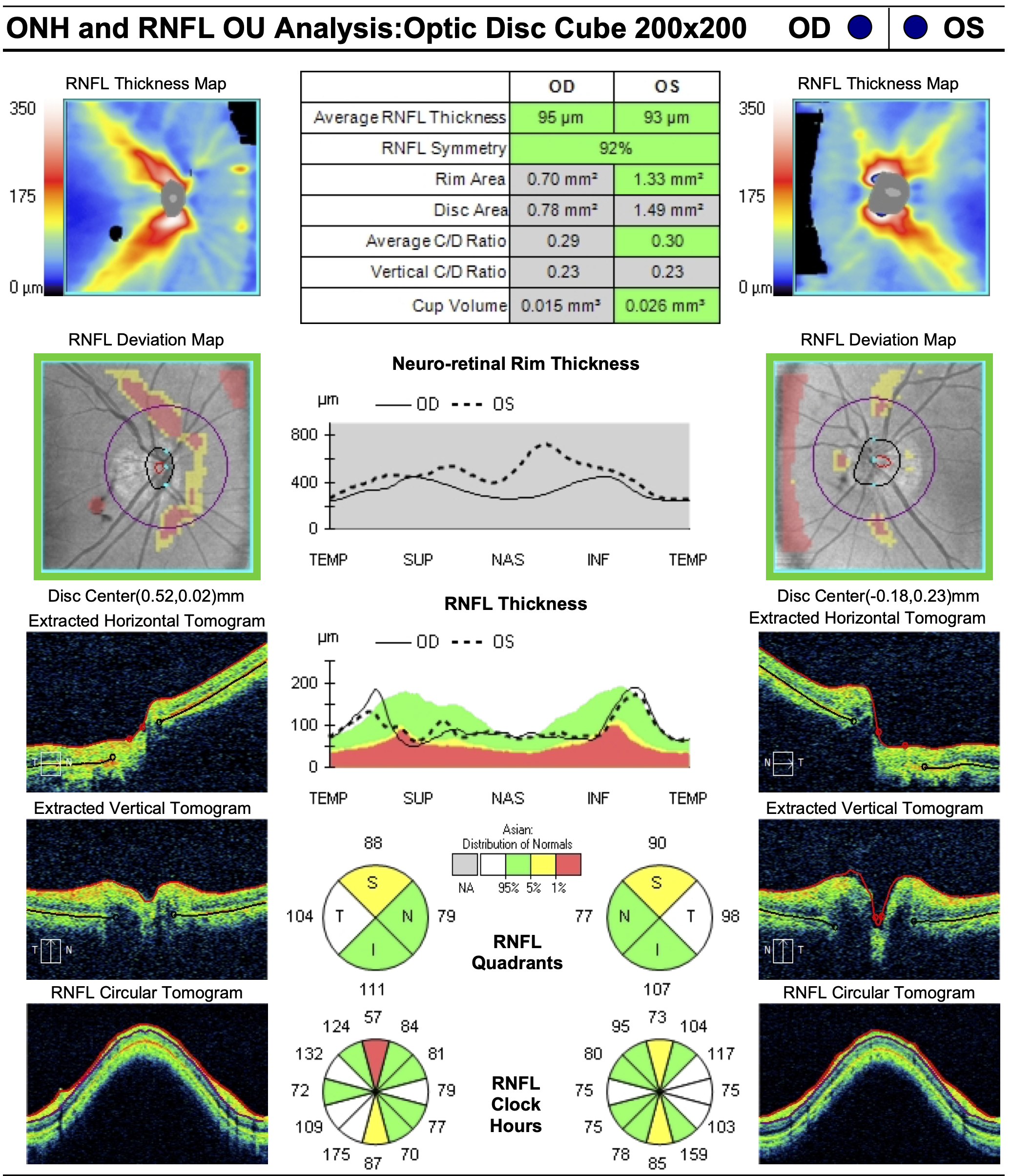


B.


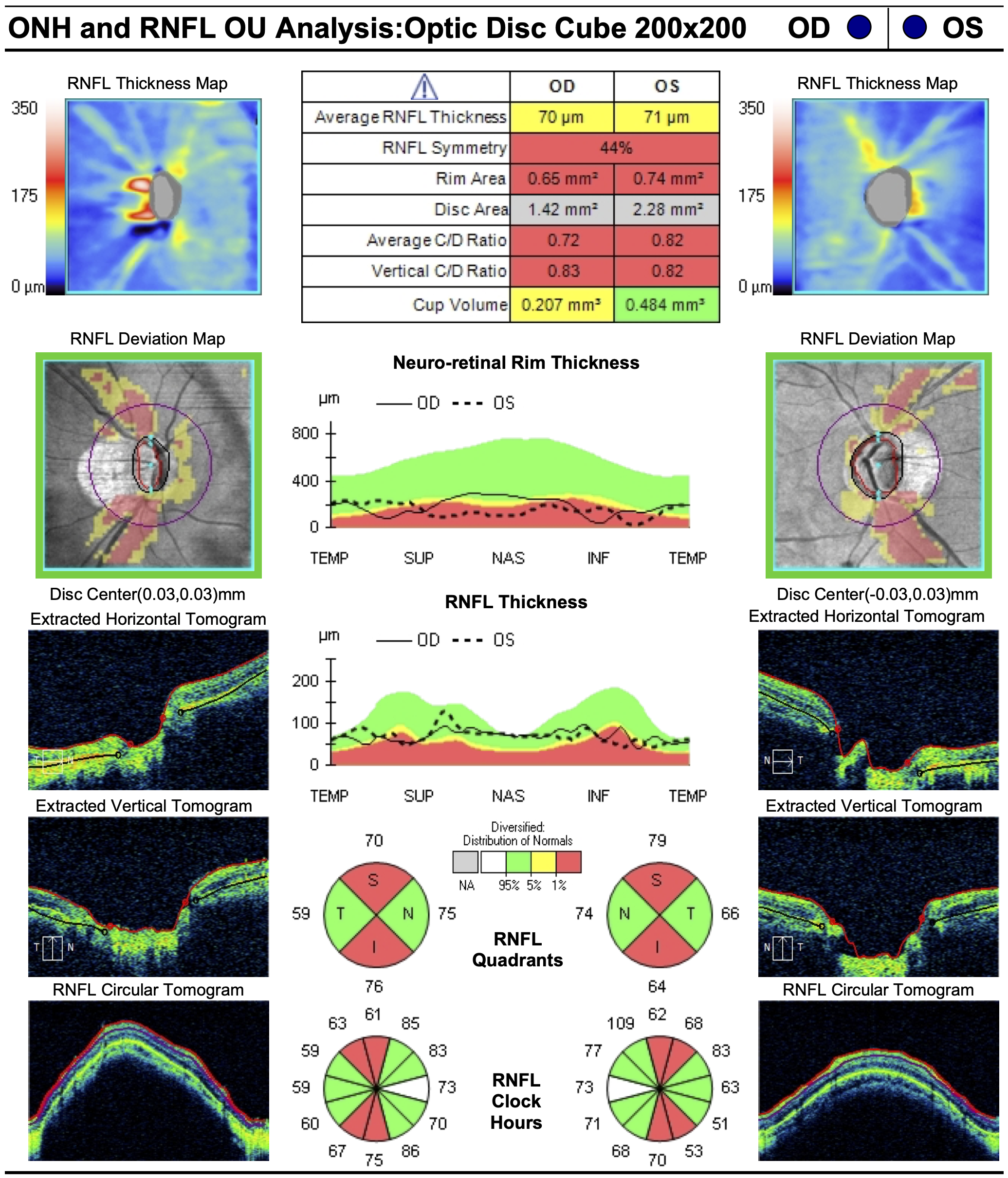


**Supplementary Figure 2.** Examples of SDOCT paired *en face* images and volumetric scans with or without myopic features (MF). (a) The *en face* image and volumetric scans with MF due to peripapillary atrophy (PPA) and optic disc tilting. (b) The *en face* image and volumetric scans without any MF.

**
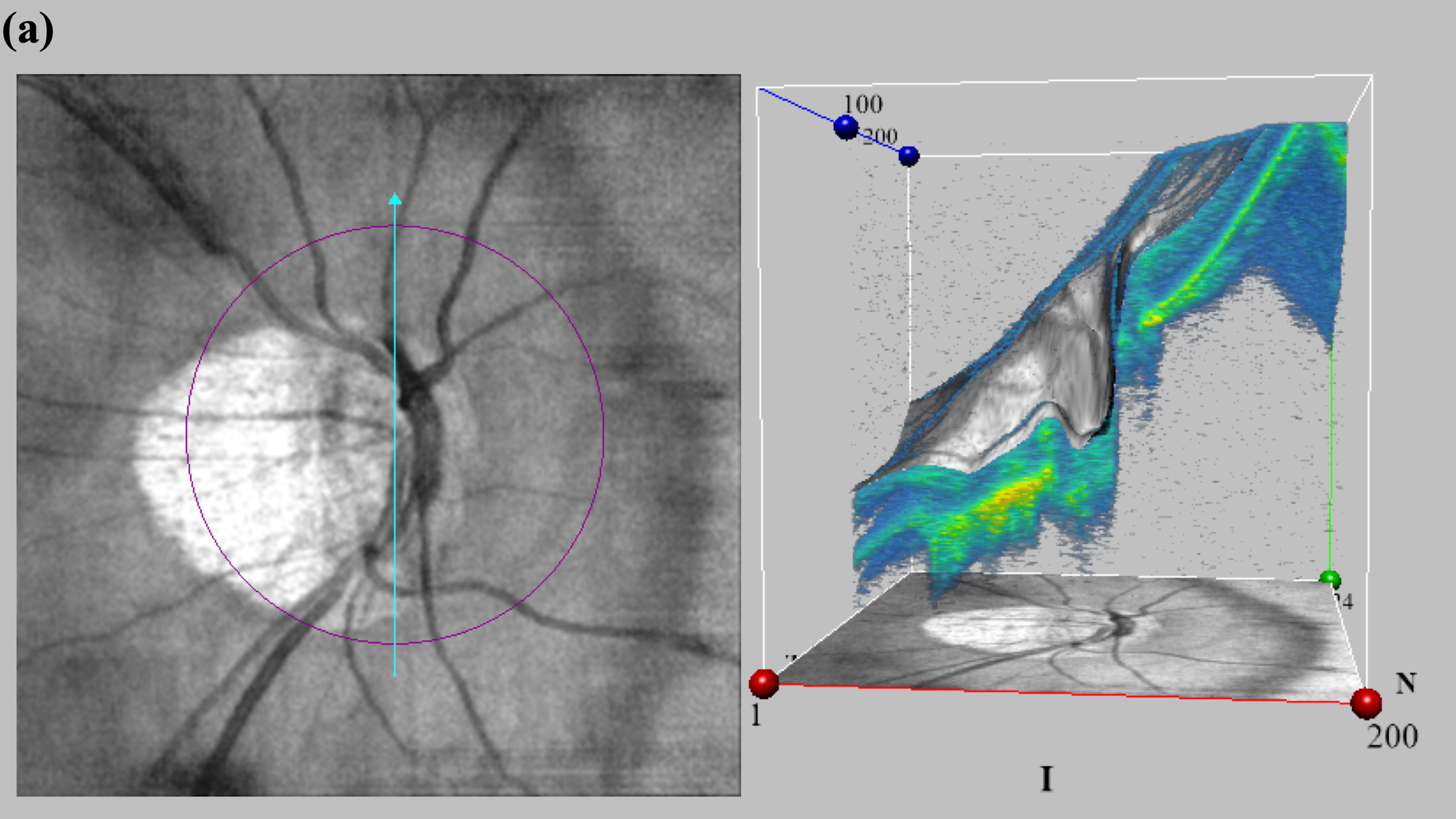

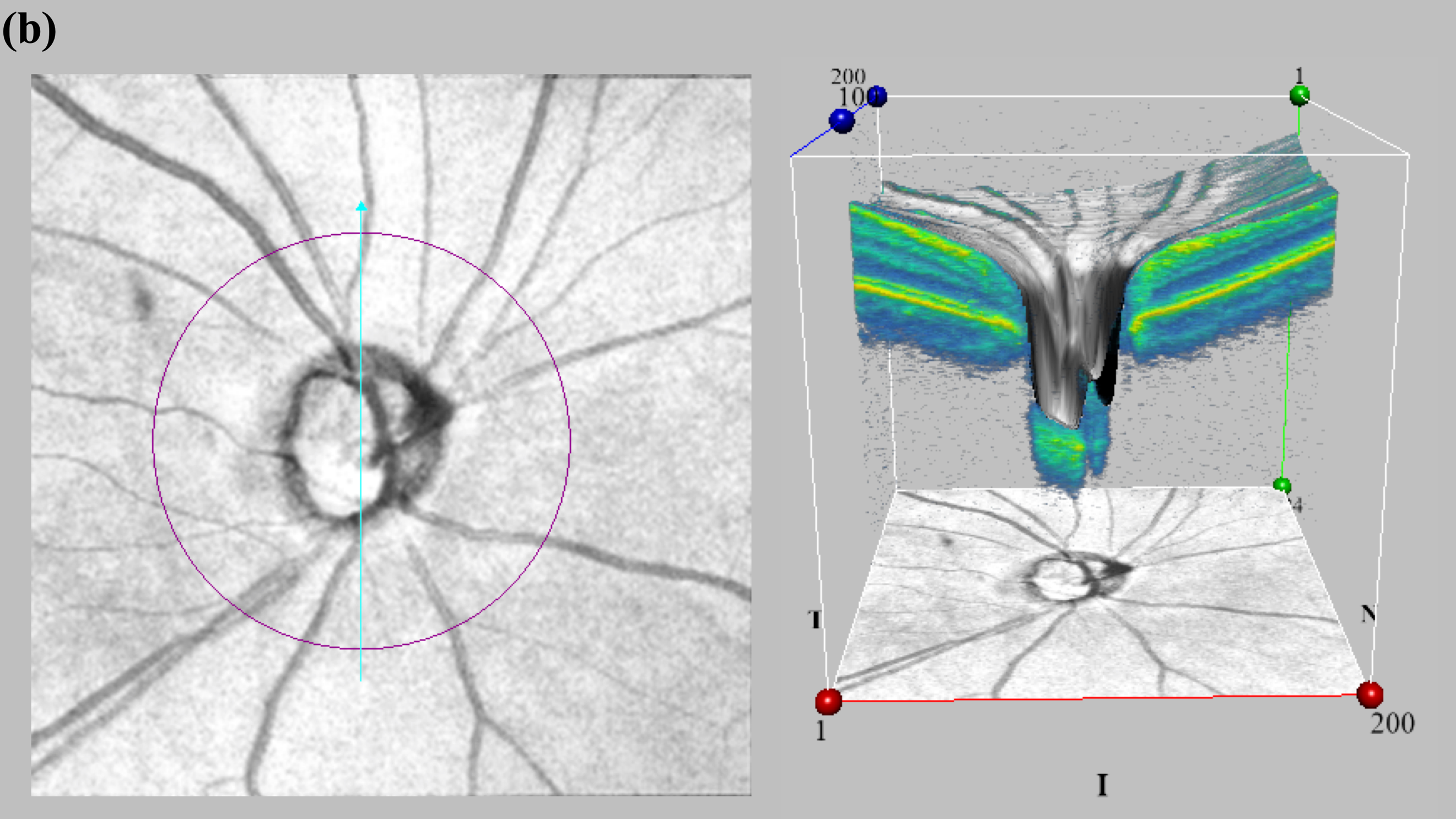
**

**Supplementary Figure 3.** The area under the receiver operating characteristic curve (AUROC) values of the multi-task DL model in internal validation and external testing for myopic features detection.

**
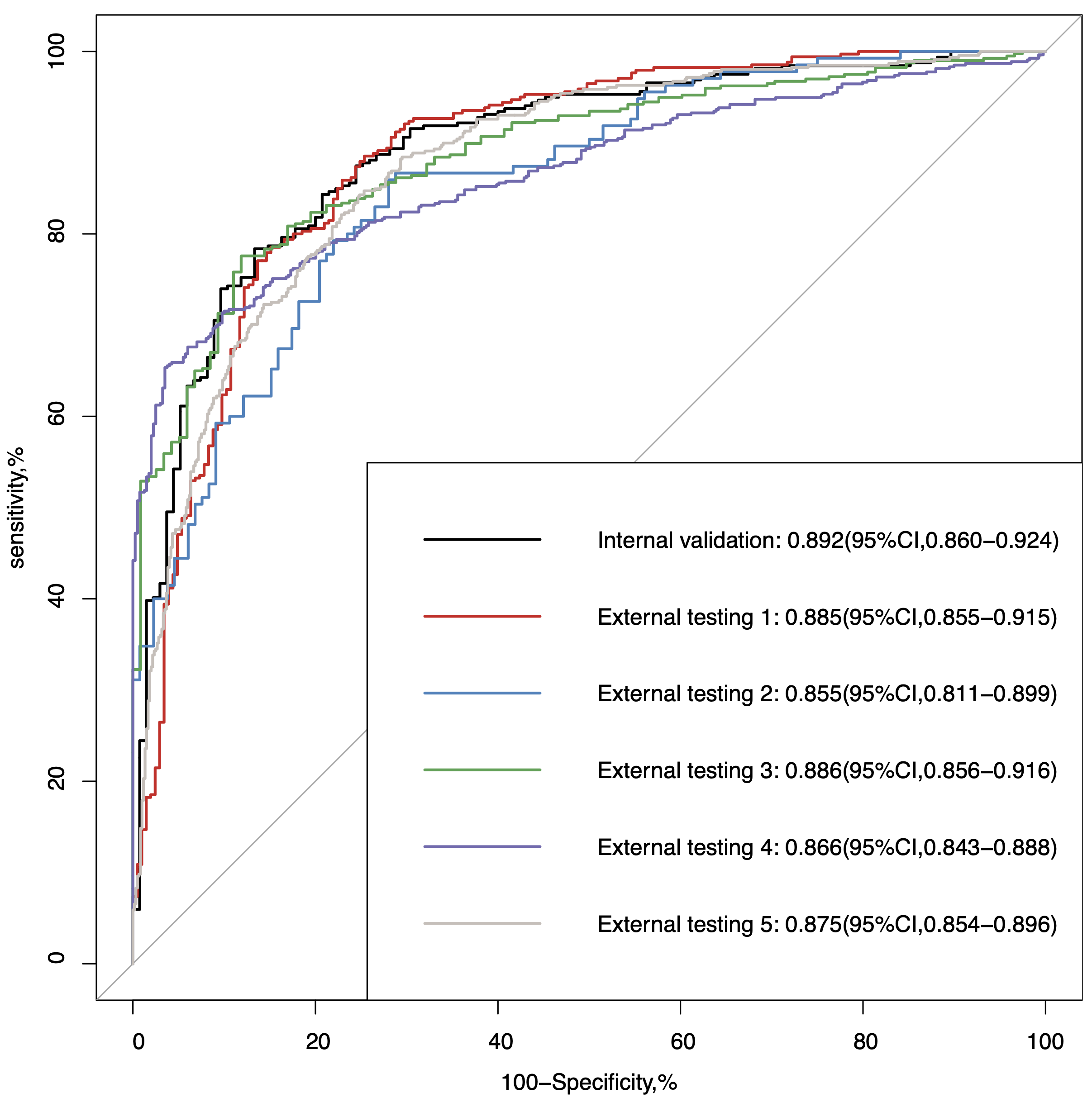
**

**Supplementary Figure 4.** The training-tuning curve showed that the multi-task 3D deep learning model converged approximately around the 30^th^ epoch and kept stable without significant oscillation after the 50^th^ epoch.


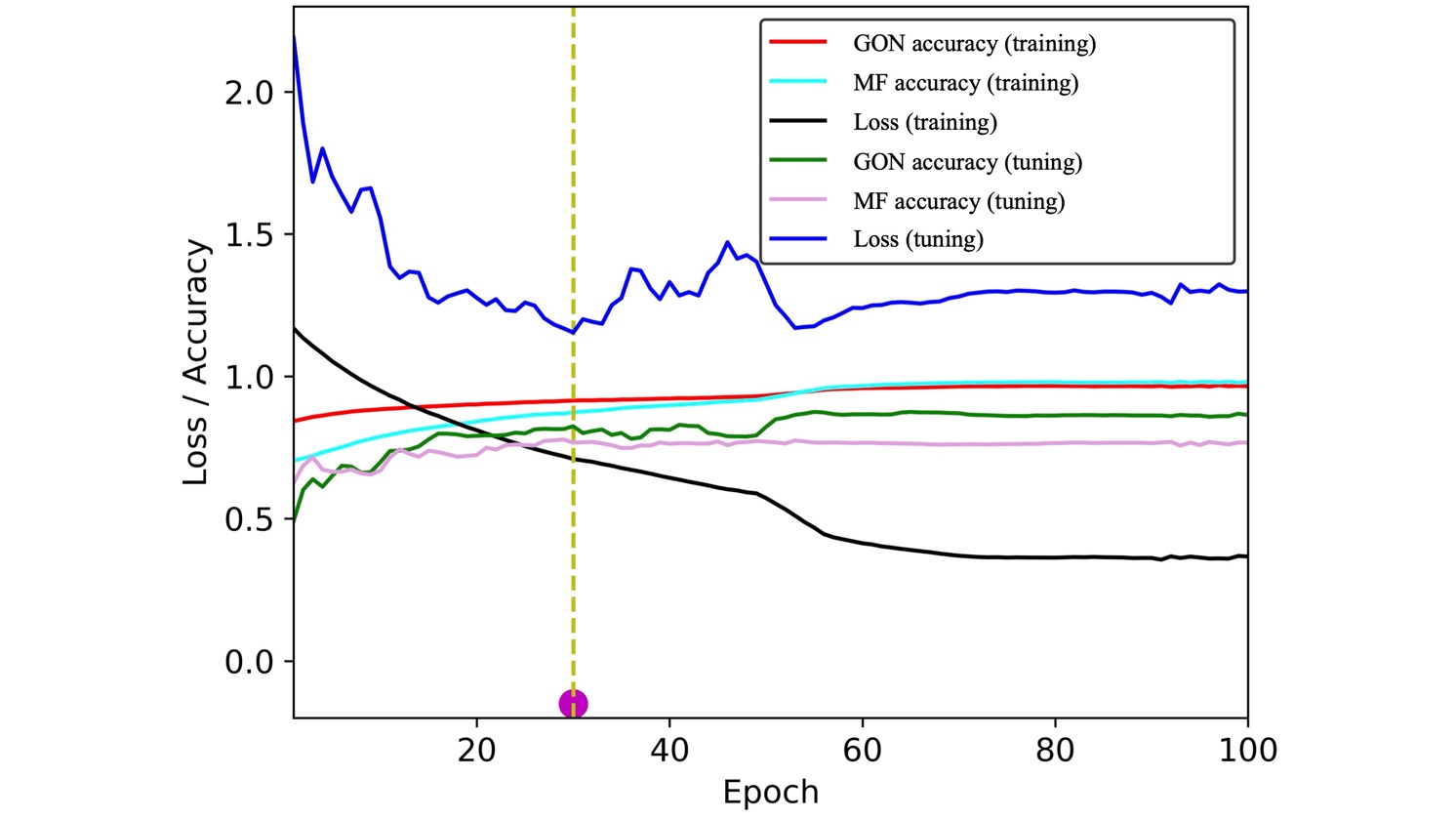

Supplement: Supplementary file 1 [file Table_1.DOCX]
